# Evaluation of the Access Bio CareStart rapid SARS-CoV-2 antigen test in asymptomatic individuals tested at a community mass-testing program in Western Massachusetts

**DOI:** 10.1038/s41598-022-25266-3

**Published:** 2022-12-09

**Authors:** Sara Suliman, Wilfredo R. Matias, Isabel R. Fulcher, Francisco J. Molano, Shannon Collins, Elizabeth Uceta, Jack Zhu, Ryan M. Paxton, Sean F. Gonsalves, Maegan V. Harden, Marissa Fisher, Jim Meldrim, Stacey Gabriel, Molly F. Franke, Deborah T. Hung, Sandra C. Smole, Lawrence C. Madoff, Louise C. Ivers

**Affiliations:** 1 Division of Rheumatology, Inflammation and Immunity, Brigham and Women’s Hospital, Harvard Medical School, Boston, MA USA; 2 Division of Experimental Medicine, Department of Medicine, Zuckerberg San Francisco General Hospital, University of California San Francisco, San Francisco, CA USA; 3 Division of Infectious Diseases, Massachusetts General Hospital, Boston, MA USA; 4 Center for Global Health, Massachusetts General Hospital, Boston, MA USA; 5 Department of Global Health and Social Medicine, Harvard Medical School, 641 Huntington Avenue, Boston, MA 02115 USA; 6 Harvard Data Science Initiative, Cambridge, MA USA; 7 Faculty of Medicine, University of Antioquia, Medellín, Antioquia Colombia; 8 Board of Health, City of Holyoke, Holyoke, MA USA; 9 Broad Institute of MIT and Harvard, 415 Main St., Cambridge, MA USA; 10 Massachusetts Department of Public Health, Jamaica Plain, MA USA; 11 Harvard Global Health Institute, Cambridge, MA USA

**Keywords:** Infectious diseases, Immunology, Microbiology, Diseases, Biomarkers, Diagnostic markers

## Abstract

Point-of-care antigen-detecting rapid diagnostic tests (RDTs) to detect Severe Acute Respiratory Syndrome Coronavirus 2 (SARS-CoV-2) represent a scalable tool for surveillance of active SARS-CoV-2 infections in the population. Data on the performance of these tests in real-world community settings are paramount to guide their implementation to combat the COVID-19 pandemic. We evaluated the performance characteristics of the CareStart COVID-19 Antigen test (CareStart) in a community testing site in Holyoke, Massachusetts. We compared CareStart to a SARS-CoV-2 reverse transcriptase quantitative polymerase chain reaction (RT-qPCR) reference, both using anterior nasal swab samples. We calculated the sensitivity, specificity, and the expected positive and negative predictive values at different SARS-CoV-2 prevalence estimates. We performed 666 total tests on 591 unique individuals. 573 (86%) were asymptomatic. There were 52 positive tests by RT-qPCR. The sensitivity of CareStart was 49.0% (95% Confidence Interval (CI) 34.8–63.4) and specificity was 99.5% (95% CI 98.5–99.9). Among positive RT-qPCR tests, the median cycle threshold (Ct) was significantly lower in samples that tested positive on CareStart. Using a Ct ≤ 30 as a benchmark for positivity increased the sensitivity of the test to 64.9% (95% CI 47.5–79.8). Our study shows that CareStart has a high specificity and moderate sensitivity. The utility of RDTs, such as CareStart, in mass implementation should prioritize use cases in which a higher specificity is more important, such as triage tests to rule-in active infections in community surveillance programs.

## Introduction

The Coronavirus disease 2019 (COVID-19) pandemic caused by the Severe Acute Respiratory Syndrome Coronavirus 2 (SARS-CoV-2) is the most significant infectious disease pandemic in the last century^1,2^. In addition to preventive measures such as social distancing, mask wearing, and vaccination, pillars of pandemic control rely on tools to rapidly identify cases and monitor transmission^3^. Molecular testing methods based on reverse transcription quantitative polymerase chain reactions (RT-qPCR) remain the backbone of many testing programs globally^4^. However, RT-qPCR-based testing is heavily influenced by supply chain restrictions, need for trained personnel and central laboratories, and relatively long turnaround times, particularly in resource-constrained settings^5^. Therefore, it is still challenging to scale up RT-qPCR tests for population surveillance and the timely detection of the large proportion of asymptomatic SARS-CoV-2-infected carriers^6^. Rapid detection of SARS-CoV-2 infected individuals allows for faster clinical intervention and implementation of public health measures such as isolation and contact-tracing, to prevent forward transmission^7^.

Rapid antigen-detecting diagnostic tests (RDTs) for COVID-19, many of which can yield actionable results in turnaround times often below 20 min, require little laboratory capacity, and can be performed easily by non-laboratory personnel^8^. Furthermore, decentralized access to RT-qPCR testing remains sparse in resource-constrained communities^9^. The low cost of antigen-detecting RDTs, short turnaround times and ease of use make them excellent candidates to increase their accessibility for large-scale implementation in varied community settings^10^.

Since the pandemic’s onset, several antigen-detecting RDTs have been developed for the detection of SARS-CoV-2^11^. Many of the antigen-detecting RDTs received Emergency Use Authorization (EUA) approvals by the Food and Drug Administration (FDA)^12^. Indeed, over the last year, rapid diagnostic tests have become more widely used for the diagnosis of COVID-19 in diverse settings outside the hospital, including at-home testing. However, evaluations to receive EUA were performed by demonstrating accuracy in symptomatic individuals only^13^. This narrow indication for antigen-detecting RDTs raises their limited utility in detecting SARS-CoV-2 infection in asymptomatic carriers. In fact, several independent evaluations demonstrate the decreased sensitivity of antigen-detecting RDTs in asymptomatic RT-qPCR positive individuals compared to those with symptoms^14–17^. In the United States, studies thus far have focused on 3 RDTs: Quidel Sofia^8,18^, BD Veritor^13,19^ and Abbott BinaxNOW^15,16,20^. On March 31st, 2021, the FDA also authorized these tests for home use, raising concerns about misinterpretation of false negative results^21^. Therefore, evidence to establish their performance characteristics to guide their implementation in real-world settings is even more urgent now.

In this study, we evaluated the Access Bio CareStart COVID-19 RDT (CareStart), a chromatographic antigen-detecting lateral flow immunoassay that received EUA by the FDA on October 8th, 2020^12,17^. We evaluated CareStart in asymptomatic and mildly symptomatic individuals presenting for routine testing at one of the ‘Stop the Spread’ free community testing sites in Holyoke, Massachusetts^22^. Public health messaging for testing at these community testing sites targeted asymptomatic individuals. We evaluate the sensitivity, specificity, and positive (PPV) and negative predictive values (NPV) as a function of different prevalence scenarios.

## Methods

### Study population and ethical approval

This was a prospective evaluation using convenience sampling of asymptomatic and mildly symptomatic individuals presenting for routine testing for COVID-19. The study was performed between January 6 and February 26, 2021, at the Holyoke “Stop the Spread” walk-up testing site, a free Massachusetts public testing program, which targets asymptomatic individuals^22^. The testing site opened three days a week. Individuals who presented to the site during testing hours were approached by our research staff who explained the nature of the study, risks, benefits, and answered any questions before inviting individuals to participate in the study. Informed verbal consent, in lieu of written consent, was obtained and documented by the research staff from participants standing in testing lines to collect a second anterior nasal swab as well as from guardians of minors below 18 years of age, from whom verbal assent was also obtained. The participants were treated in accordance with Good Clinical Practice guidelines and the Declaration of Helsinki. The study protocol was approved by the Partners Institutional Review Board (Protocol ID: 2020P003892).

### Study intake and data collection

After enrollment in the study, our study staff implemented an intake questionnaire capturing information on participant demographics, presence or absence of symptoms based on case definitions from the Council for State and Territorial Epidemiologists^23^: cough, sore throat, chills, shortness of breath, fever, muscle aches or soreness, nausea, vomiting or diarrhea, decreased sense of smell or taste, loss of appetite, general weakness or fatigue, or headaches. The survey also captured prior COVID-19 testing and potential exposures. Each test was assigned a unique anonymous ID. Data collected was inputted into a secure Research Electronic Data Capture (REDCap) database on encrypted tablets. We used the demographic information and specimen numbers to match the RDTs result with the RT-qPCR data collected at the Broad Institute Clinical Research Sequencing Platform (CRSP) as performed in other studies^15,24^.

### Swab collection procedure

The sample was collected at the city testing site by personnel who had received a brief training on performance of the RDT but were not trained health care providers or diagnostic specialists. We used dry anterior nasal (AN) swabs: Puritan 6″ Sterile Standard Foam Swab with Polystyrene Handle (Puritan, Guilford). Both anterior nares were swabbed 2 times (5 rotations in each nostril), once for RT-qPCR testing and once for the RDT sample. For practical reasons, the swabs for RT-qPCR and RDT were not always collected in the same order. Both samples were placed inside closed test tubes. The RT-qPCR sample was transported to the Broad Institute at the Massachusetts Institute of Technology. The second anterior nasal swab sample was transported to a nearby testing station and the RDT was performed within an hour of sample collection. The RT-qPCR testing results were interpreted according to the publicly available rubric for the Broad Institute COVID-19 testing program: https://sites.broadinstitute.org/safe-for-school/result-code-information. Briefly, the assay is a multiplexed RT-qPCR assay, which runs up to 40 cycles. The assay targets the N1 and N2 genes, using the CDC primers, with an RNase P (RP) gene as an internal control gene for test validity^25^. Any cycle threshold (Ct) value for either N1 or N2 below 40 is considered a positive result, which is how we define SARS-CoV-2-positive individuals to benchmark the performance of the CareStart RDT.

### Rapid test procedure

The CareStart device came with instructions for use and diagrams. The study staff received a one-hour training prior to the study and practiced the RDT on positive and negative control samples provided in the kit. One operator performed the test at a workstation following the CareStart manufacturer’s instructions for use (IFU)^26^, took pictures of the tests, read the result as positive or negative, and captured into the electronic data entry forms. Participants with a positive RDT were contacted by phone per request from the Department of Public Health within a twenty-four-hour period, informed of their result, and advised to isolate until they received their RT-qPCR result.

### Reference RT-qPCR standard

The gold standard reference used was the SARS-CoV-2 RT-qPCR laboratory developed test through the Broad Institute CRSP, which is approved by the FDA under EUA. The test provides two cycle threshold (Ct) values, one for the nucleocapsid (N2) gene, and one for an internal positive control RNaseP gene. We compared the sensitivity of CareStart against both the qualitative binary RT-qPCR results and the Ct values of the N2 gene amplification reaction, as previously described^17^.

### Statistical analyses

We calculated sensitivity, specificity, PPV and NPV of the RDT from 2 × 2 contingency tables using RT-qPCR as the gold standard reference. Sensitivity and specificity were further stratified and compared by presence of symptoms and quantitative Ct values. Median Ct values were compared using the non-parametric unpaired Mann–Whitney *U* test. 95% Pearson-Clopper confidence intervals (CI) were calculated for sensitivity and specificity estimates. Since RDTs have been reported to have high accuracy among symptomatic individuals^8,15–17^, we also tested whether presence of symptoms would increase the sensitivity of the CareStart RDT. Statistical analyses were conducted using R V3.6.0 (R Core Team 2020).

## Results

**Figure 1:**
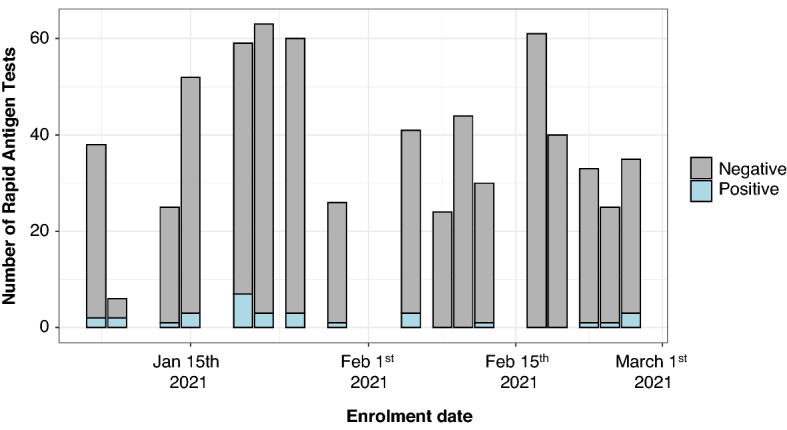
Number of CareStart rapid antigen test administered by date (n = 666). The bar colors reflect the results of the rapid tests on different days.

**Figure 2:**
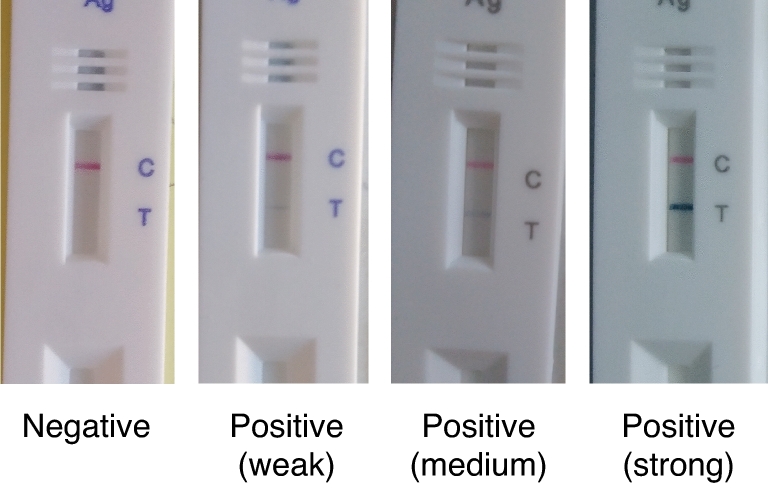
Examples of images of CareStart rapid test showing variable band intensities.

We performed 666 CareStart RDTs from participants who provided verbal consent at the walk-up testing site. Of these, 4 tests were excluded because test vial caps were malformed and the operator was unable to load the RDT, resulting in 662 tests included for analysis. The 662 tests performed were comprised of 588 unique participants. (Tables 1, 2 and Fig. 1). 60 participants by chance received more than one test, with a total of 75 tests performed in addition to the first test per participant (Supplementary Table 1). Among the 588 participants, 51.9% were residents from Holyoke, as identified by their residential zip codes. Just over half the participants (51.9%) identified as female. The mean age was 38.1, and 44.7% of participants identified as Hispanic or LatinX (Table 1). The study staff evaluated the usability of the CareStart devices. All tests showed a positive control band, indicating they were valid. The RDT procedures involved immersing a swab into a vial consisting of extraction buffer, subsequently, the swab was taken away and the cap was used to close the vial. A few drops of the specimen solution were applied to the test device. Of the valid tests, we noted variable band intensities (Fig. 2). The positive test line was sometimes so faint that a flashlight was necessary to see it.

**Table 1:** Demographics of unique study participants who enrolled in the CareStart Rapid Antigen Test evaluation at the Stop the Spread COVID-19 testing site in Holyoke, Massachusetts.

Demographics	Always negative (N = 558)	Ever positive (N = 30)	Overall (N = 588)
**Age (years)**			
Mean (SD)	38.2 (17.9)	37.4 (15.4)	38.1 (17.8)
Median [Min, Max]	36 [1, 85]	34.5 [16, 75]	36 [1, 85]
**Sex**			
Female	294 (52.7%)	11 (36.7%)	305 (51.9%)
Male	236 (42.3%)	14 (46.7%)	250 (42.5%)
Non-binary	4 (0.7%)	0 (0%)	4 (0.7%)
Prefer not to answer	24 (4.3%)	4 (13.3%)	28 (4.7%)
Not listed	0 (0%)	1 (3.3%)	1 (0.2%)
**Lives in Holyoke zip code**			
Yes	292 (52.3%)	13 (43.3%)	305 (51.9%)
No	266 (47.7%)	17 (56.7%)	283 (48.1%)
**Race/ethnicity category**			
Hispanic or LatinX	248 (44.4%)	15 (50.0%)	263 (44.7%)
White Non-Hispanic	262 (47%)	14 (46.7%)	276 (46.9%)
Asian Non-Hispanic	5 (0.9%)	0 (0%)	5 (0.9%)
Black or African American Non-Hispanic	15 (2.7%)	0 (0%)	15 (2.5%)
Native Hawaiian or Other Pacific Islander Non-Hispanic	1 (0.2%)	0 (0%)	1 (0.2%)
Other Non-Hispanic	8 (1.4%)	0 (0%)	8 (1.4%)
Two or more races Non-Hispanic	4 (0.7%)	0 (0%)	4 (0.7%)
Prefer not to answer	15 (2.7%)	1 (3.3%)	16 (2.7%)
**Repeat tester**			
No	506 (90.7%)	22 (73.3%)	528 (89.8%)
Yes	52 (9.3%)	8 (26.7%)	60 (10.2%)

**Table 2:** Tester symptoms, exposure history and prior COVID-19 testing per each CareStart testing occurrence, including repeated tests from the same participants.

	Negative (N = 631)	Positive (N = 31)	Overall (N = 662)
**Any symptoms**			
No	554 (87.8%)	18 (58.1%)	572 (86%)
Yes	77 (12.2%)	13 (41.9%)	90 (14%)
**Exposure**			
Confirmed or suspected	196 (31.1%)	17 (54.8%)	213 (32.2%)
No exposure	433 (68.6%)	14 (45.2%)	447 (67.5%)
Did not answer	2 (0.3%)	0 (0%)	2 (0.3%)
**Prior COVID-19 test**			
No	110 (17.4%)	9 (29.0%)	119 (18.0%)
Yes	519 (82.3%)	22 (71.0%)	541 (81.7%)
Missing	2 (0.3%)	0 (0%)	2 (0.3%)

To determine the accuracy of the CareStart RDT, we calculated the concordance between the RDT and RT-qPCR (Table 3). Thirty-one RT-qPCR tests were excluded from the analysis because the sample was unsatisfactory for processing, or testing yielded inconclusive results (detection of one of the two viral probes). This testing in real-world settings would have prompted a recommendation for re-testing. Using all RT-qPCR values below 40 as a positive reference, the sensitivity of the CareStart RDT was 48.1% (95% CI 34.0–62.4%), while the specificity was 99.0% (95% CI 97.8–99.6%) (Table 4). Of the 662 visits, participants reported presence of symptoms 90 (14.0%) times (Table 2). Cough was the most reported (n = 38, 5.7%) symptom, while loss of smell or taste, a more specific COVID-19 symptom, was only reported in 18 RDTs (2.7%) (Supplementary Table 2). Participants who tested positive for the CareStart RDT were more likely to report at least one symptom compared to participants that tested negative (41.9% vs. 12.2%; Chi-square *p* < 0.0001) (Table 2). Due to the limited sample size, we only stratified individuals tested by presence (n = 90) or absence (n = 572) of symptoms to test the CareStart RDT accuracy as a function of symptoms. The sensitivity of CareStart RDT in symptomatic individuals was 46.4% (95% CI 27.5–66.1%), and the specificity was 100% (95% CI 95–100%) (Supplementary Table 3A,B). In asymptomatic individuals, the sensitivity of the CareStart RDT was 52.2% (95% CI 30.6–73.2%), and the specificity was 99.4% (95% CI 98.3–99.9%) (Supplementary Table 3C,D). Sensitivity and specificity did not significantly differ between symptomatic and asymptomatic individuals (*p* = 0.781 for sensitivity; *p* > 0.999 for specificity).

**Table 3:** Concordance between CareStart test results and RT-qPCR test results.

Result of N gene RT-qPCR	Negative (N = 580)	Positive (N = 51)	Overall (N = 631)
**CareStart test result**			
Negative	577 (99.5%)	26 (51%)	603 (95.6%)
Positive	3 (0.5%)	25 (49%)	28 (4.4%)

**Table 4:** Performance characteristics of CareStart test results benchmarked against the RT-qPCR gold standard.

	n	Total tests	Performance characteristic	Estimate (%)	95% Confidence interval
**Rapid test results**					
Positive	25	51	Sensitivity	49.0	(34.8–63.4%)
Negative	577	580	Specificity	99.5	(98.5–99.9%)

**Figure 3:**
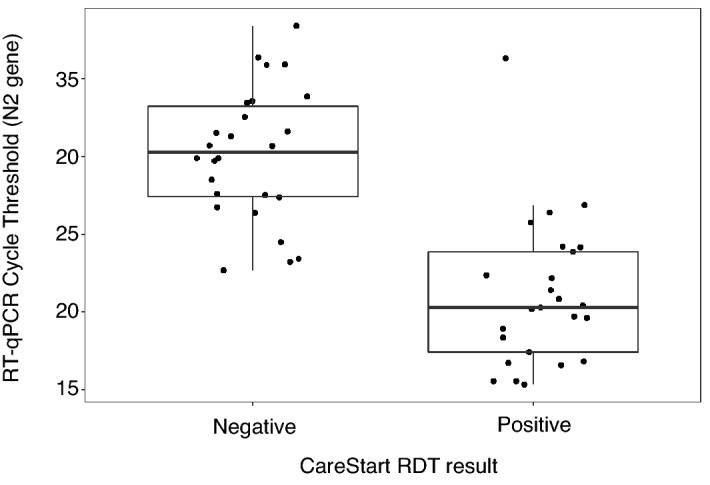
N2 gene RT-qPCR Cycle threshold (Ct) values corresponding to positive and negative CareStart rapid antigen test results for all RT-qPCR positive samples (n = 52).

Next, we used Ct values for amplification of the N2 target as a proxy for viral load, where higher Ct values reflected low viral loads, as previously reported^27^. The Ct values of samples recorded as negative using the CareStart RDT were significantly higher than positive counterparts (Mann Whitney *U* *p* value < 0.0001, Fig. 3). Therefore, we also performed a subset analysis where we only considered samples with a Ct < 30 as positive (Table 5). Using this cut-off, the CareStart RDT sensitivity and specificity were 64.9% (95% CI 47.5–79.8%) and 99.3% (95% CI 98.3–99.8%), respectively (Table 6). Although the CareStart RDT EUA does not indicate a specific Ct threshold for the positivity of the comparator RT-qPCR^26^, these data suggest that applying a more stringent Ct value threshold moderately improves the sensitivity of the CareStart RDT.

**Table 5:** CareStart test results compared to RT-qPCR using Ct positivity threshold of < 30.

CareStart results	Negative (Ct > = 30) N = 594	Positive (Ct < 30) N = 37	Overall N = 631
Negative	590 (98.8%)	13 (34.2%)	603 (95.6%)
Positive	4 (0.7%)	24 (63.2%)	28 (4.4%)

**Table 6:** CareStart test result sensitivity and specificity using Ct positivity threshold of < 30.

Type	n	N	Estimate (%)	95% CI
Sensitivity	24	37	64.9	47.5–79.8%
Specificity	590	594	99.3	98.3–99.8%

**Figure 4:**
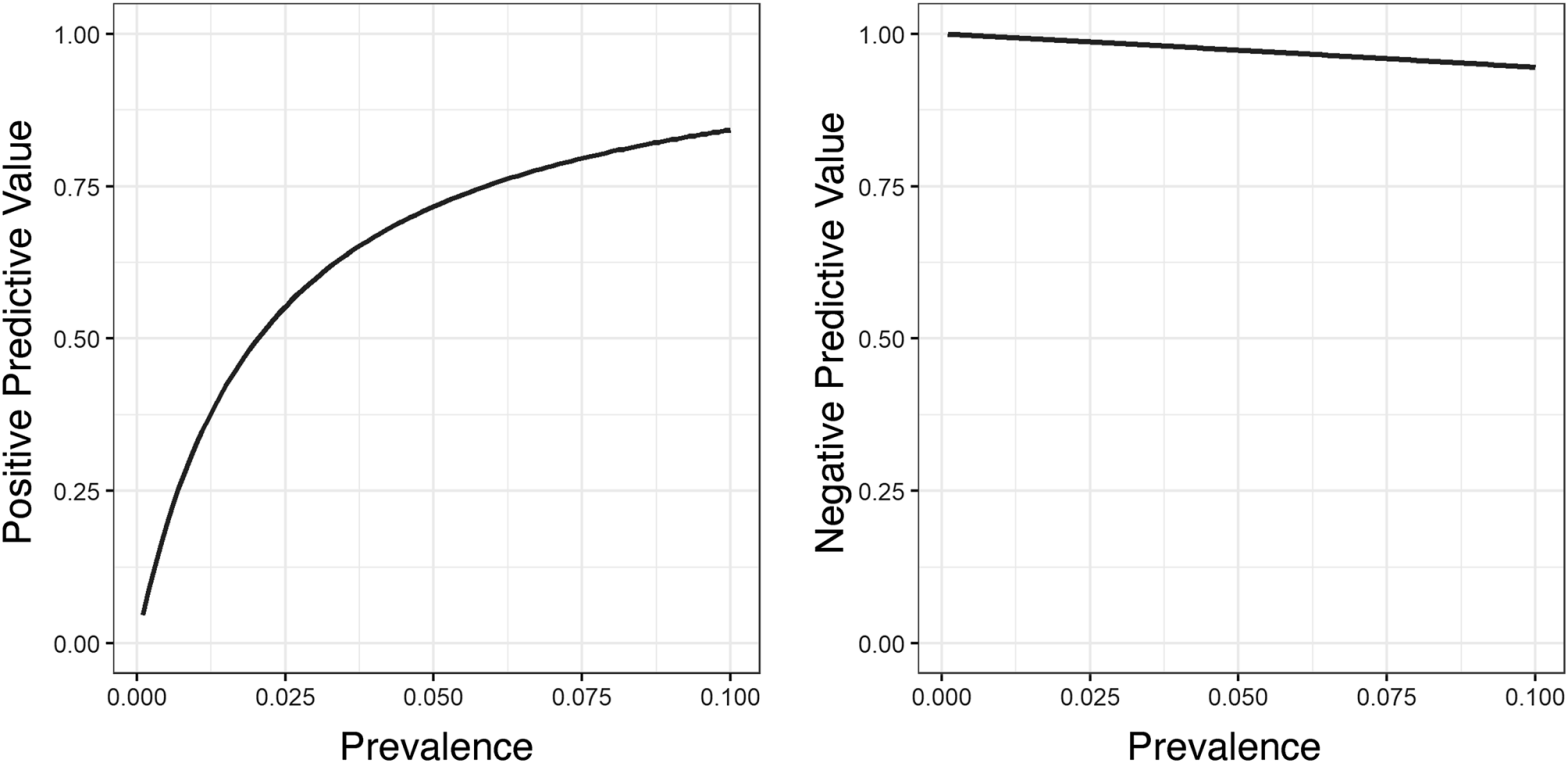
Calculated positive (left) and negative predictive values (right) based on the CareStart performance characteristics and different prevalence estimates of SARS-CoV-2 infections.

Positive and negative predictive values of diagnostic tests depend on the prevalence of infections in a population, where a higher prevalence increases the PPV at the expense of the NPV^28^. We calculated the PPV and NPV values as a function of prevalence rates up to 10%, where the PPV steeply dropped in prevalence rates lower than 5% (Fig. 4). At a sensitivity of 49% and specificity of 99.5% (Table 4), the PPV of CareStart was 49.7% at a SARS-CoV-2 infection prevalence of 1%, and 91.6% at a prevalence of 10%. In contrast, the NPV was 99.5% at a prevalence of 1%, and 94.6% at a prevalence of 10%.

**Figure 5:**
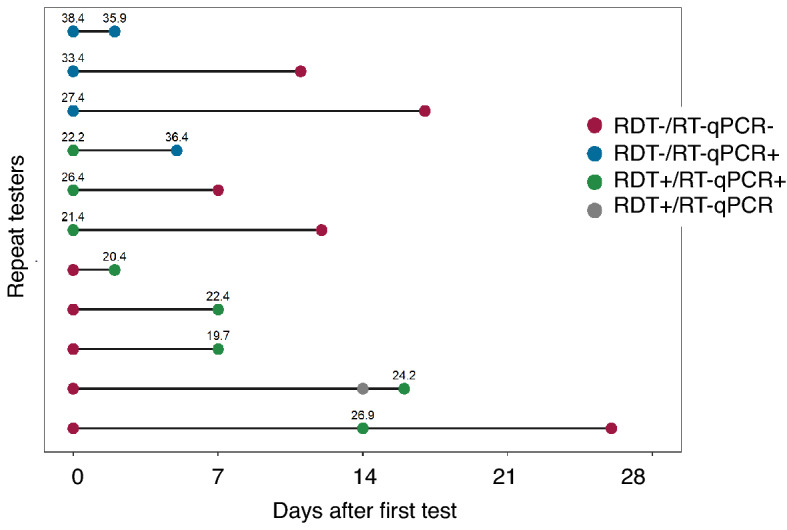
Individuals who enrolled in the study multiple times and had at least one positive gold standard RT-qPCR reference (n = 11). The point colors reflect the different combinations of RT-qPCR and CareStart rapid test results. The numbers above the point correspond to Ct values of the RT-qPCR.

Finally, our cohort included individuals who presented to the testing site multiple times, who had at least one positive RT-qPCR test result. Therefore, we performed an exploratory analysis of their longitudinal test results (Supplementary Table 1 and Fig. 5). We enrolled 5 participants who converted from a negative to positive on RT-qPCR tests, all of which were accurately detected as positive by the RDT. Two participants with both positive RT-qPCR and RDT test results reverted to negative test results on both platforms. However, one participant converted from a positive to negative RDT test result but was detected as positive by the RT-qPCR on the second test, which was conducted in less than a week.

## Discussion

Antigen-detecting RDTs provide a scalable and affordable alternative to molecular tests for the diagnosis of SARS-CoV-2 infection^29^. In this study, we present a prospective evaluation of the CareStart antigen-detecting RDT for SARS-CoV-2 detection in a real-world, walk-up community COVID-19 testing site in Holyoke, Western Massachusetts, a region experiencing disparities in testing that could be addressed by the scale-up of validated, affordable antigen-detecting RDTs such as CareStart^30^.

Compared to a RT-qPCR-based test performed on anterior nasal swabs, we found a much lower sensitivity (49.0%) than what was reported in the FDA package insert (87.2%), which was restricted to 39 symptomatic individuals within 5 days of symptom onset^26^. However, our measured sensitivity was consistent with the reported sensitivity of CareStart in asymptomatic individuals from a study at Lawrence General Hospital in Massachusetts where the estimated sensitivity of CareStart was 51.4%^17^. Compared to the Abbott BinaxNOW RDT, which has been validated in several studies including a recent study in Massachusetts, the sensitivity of the CareStart RDT was lower overall and when using a Ct positivity cutoff of ≤ 30^15^. The specificity of both tests was comparable at nearly > 99%. Consistent with several studies, the sensitivity of antigen detecting RDTs was modest in individuals with no or mild symptoms^8,14–16,20^. Given that presymptomatic and asymptomatic transmission is an important component of the pandemic^31^, implementation of antigen-detecting RDTs needs to weigh the benefits of rapid detection of SARS-CoV-2-infected individuals with the lower sensitivity of these tests in asymptomatic carriers^29^. On the other hand, the high specificity of these tests reduces the probability of false positive SARS-CoV-2 test results that could lead to restrictions and inconveniences that interfere with the livelihood of these individuals.

The RT-qPCR cycle threshold (Ct), a proxy for lower viral load, as reported in other evaluations^15,17,32^, had a clear impact on the sensitivity of the CareStart RDT. Concordant results showed lower Ct values whereas discordant results had higher Ct values. Consistent with this, the CareStart RDT sensitivity improved with a RT-qPCR positivity Ct cut-off of < 30. These data suggest that the CareStart RDT positivity suggested a state of higher viral load, which correlates with infectivity of cells in vitro^33^ and likely transmissibility. The viral load at the time of testing depends on multiple factors including host vaccination status, host immunity, viral variants and replication kinetics, local epidemic curves, and the method and quality of sample collection. For example^34^, individuals vaccinated with the Pfizer/BioNtech BNT162b2 vaccine with breakthrough SARS-CoV-2 infections have been reported to show higher Ct values post-vaccination than unvaccinated counterparts, which correlates with lower viral loads^35^, suggesting that vaccinated individuals with breakthrough SARS-CoV-2 infections would be more difficult to detect by antigen-detecting RDT with similar performance characteristics to CareStart. Further studies are needed to evaluate the performance characteristics of CareStart and other RDTs in these different scenarios.

Although our sample of repeat testers was limited, it suggested that individuals who recently converted from negative to positive RT-qPCR test, i.e. recently acquired SARS-CoV-2 infection, were easily detectable by the CareStart RDT. Recently infected individuals have been shown to be more contagious^27^. Samples from these recently infected individuals had low Ct values, and were thus more likely to transmit the virus^33^. Though our data is underpowered to evaluate performance characteristics in the setting of repeat testing, this limited sample supports the usefulness of serial rapid antigen testing in detecting recent infections and guiding the implementation of containment measures^36^. This is supported by studies with other RDTs, for example, a study showing that daily testing increased the sensitivity of Quidel SARS Sofia antigen fluorescent immunoassay (FIA)^37^. The public health benefits of serial testing with RDTs should be studied further.

This study has several limitations. First, we had limited control over or ability to monitor the order by which the two bilateral nasal swabs were collected because of embedding the study in a ‘real world’ testing program. It is possible that performing the PCR swab first may decrease the available viral load for the antigen test. However, a recent evaluation of the Abbott BinaxNOW suggested that the order of swabs had little impact on the test result^20^. Second, it remains possible that we overestimated the sensitivity of the CareStart rapid test by using anterior nare swabs instead of nasopharyngeal swab samples for the qPCR reference, which may be a slightly less sensitive gold-standard compared than nasopharyngeal swabs^38,39^. Third, since rapid testing in the USA has primarily transitioned from community-based testing to over the counter direct-to-consumer testing^40^, it is important to develop tools to facilitate the interpretation of the positive predictive value of a test with low or moderate sensitivity. We previously published a tool to facilitate the interpretation of serological lateral flow assays, that translates the test accuracy and community prevalence into a PPV: https://covid.omics.kitchen/^41^, which can be applied to rapid tests. Overall, the high specificity of the test increases its utility as a rule-in triage test, since false positive results are unlikely, but it is important to emphasize that false negatives are still likely. Finally, the study was conducted in Jan-Feb 2021, when the major lineage in New England, USA were predominantly the Wuhan or alpha variants^42^. Hence, it is unclear if the CareStart RDT is less sensitive for detecting the subsequent delta or Omicron variants and sub-lineages. Finally, the study was conducted at the time when vaccination against SARS-CoV-2 was low, which may affect the generalizability of these findings. Further studies are needed to evaluate the performance characteristics of this and other SARS-CoV-2 RDTs in these different scenarios.

In conclusion, RDTs such as CareStart, can support SARS-CoV-2 testing efforts in minimally symptomatic or asymptomatic individuals. However, the impact of the limited sensitivity of these tests on their positive predictive values warrants caution. The moderate sensitivity of these tests means that some potentially infectious individuals may be classified as SARS-CoV-2-negative. Therefore, implementing RDTs for travel, home testing, or to guide re-openings of schools and workplaces should be interpreted with caution and the utility of RDTs in each of these use cases should be carefully evaluated. Furthermore, implementation studies to analyze their usefulness and acceptability by both users and providers are necessary.

## Supplementary Information

Supplementary Information.

## References

[1] Dong E, Du H, Gardner L (2020). An interactive web-based dashboard to track COVID-19 in real time. Lancet Infect. Dis..

[2] Liu K, Fang YY, Deng Y, Liu W, Wang MF, Ma JP, Xiao W, Wang YN, Zhong MH, Li CH, Li GC, Liu HG (2020). Clinical characteristics of novel coronavirus cases in tertiary hospitals in Hubei Province. Chin. Med. J. (Engl.).

[3] Ramdas K, Darzi A, Jain S (2020). ‘Test, re-test, re-test’: Using inaccurate tests to greatly increase the accuracy of COVID-19 testing. Nat. Med..

[4] Hellou MM, Gorska A, Mazzaferri F, Cremonini E, Gentilotti E, De Nardo P, Poran I, Leeflang M, Tacconelli E, Paul M (2020). Nucleic-acid-amplification tests from respiratory samples for the diagnosis of coronavirus infections: Systematic review and meta-analysis. Clin. Microbiol. Infect..

[5] Brown RCH, Kelly D, Wilkinson D, Savulescu J (2020). The scientific and ethical feasibility of immunity passports. Lancet Infect. Dis..

[6] Oran DP, Topol EJ (2020). Prevalence of asymptomatic SARS-CoV-2 infection : A narrative review. Ann. Intern. Med..

[7] Quilty BJ, Clifford S, Hellewell J, Russell TW, Kucharski AJ, Flasche S, Edmunds WJ, Centre for the Mathematical Modelling of Infectious Diseases C-wg (2021). Quarantine and testing strategies in contact tracing for SARS-CoV-2: A modelling study. Lancet Public Health.

[8] Pray IW, Ford L, Cole D, Lee C, Bigouette JP, Abedi GR, Bushman D, Delahoy MJ, Currie D, Cherney B, Kirby M, Fajardo G, Caudill M, Langolf K, Kahrs J, Kelly P, Pitts C, Lim A, Aulik N, Tamin A, Harcourt JL, Queen K, Zhang J, Whitaker B, Browne H, Medrzycki M, Shewmaker P, Folster J, Bankamp B, Bowen MD, Thornburg NJ, Goffard K, Limbago B, Bateman A, Tate JE, Gieryn D, Kirking HL, Westergaard R, Killerby M, Group CC-SL (2021). Performance of an antigen-based test for asymptomatic and symptomatic SARS-CoV-2 testing at two university campuses—Wisconsin, September–October 2020. MMWR Morb. Mortal. Wkly. Rep..

[9] Hopman J, Allegranzi B, Mehtar S (2020). Managing COVID-19 in low- and middle-income countries. JAMA.

[10] WHO. Antigen-detection in the diagnosis of SARS-CoV-2 infection using rapid immunoassays. Interim Guidance. (2020). 11 September 2020. Accessed online: https://www.who.int/publications/i/item/antigen-detection-in-the-diagnosis-of-sars-cov-2infection-using-rapid-immunoassays

[11] Dinnes J, Deeks JJ, Berhane S, Taylor M, Adriano A, Davenport C, Dittrich S, Emperador D, Takwoingi Y, Cunningham J, Beese S, Domen J, Dretzke J, Ferrante di Ruffano L, Harris IM, Price MJ, Taylor-Phillips S, Hooft L, Leeflang MM, McInnes MD, Spijker R, Van den Bruel A, Cochrane C-DTAG (2021). Rapid, point-of-care antigen and molecular-based tests for diagnosis of SARS-CoV-2 infection. Cochrane Database Syst. Rev..

[12] FDA. In Vitro Diagnostics EUAs. U S Food and Drug Administration. (2021). Published online March 8, 2021. https://www.fda.gov/medical-devices/coronavirus-disease-2019-covid-19-emergency-use-authorizations-medical-devices/vitro-diagnostics-euas. Accessed 9 March 2021.

[13] Young S, Taylor SN, Cammarata CL, Varnado KG, Roger-Dalbert C, Montano A, Griego-Fullbright C, Burgard C, Fernandez C, Eckert K, Andrews JC, Ren H, Allen J, Ackerman R, Cooper CK (2020). Clinical evaluation of BD Veritor SARS-CoV-2 point-of-care test performance compared to PCR-based testing and versus the Sofia 2 SARS Antigen point-of-care test. J. Clin. Microbiol..

[14] Landaas ET, Storm ML, Tollanes MC, Barlinn R, Kran AB, Bragstad K, Christensen A, Andreassen T (2021). Diagnostic performance of a SARS-CoV-2 rapid antigen test in a large, Norwegian cohort. J. Clin. Virol..

[15] Pollock NR, Jacobs JR, Tran K, Cranston AE, Smith S, O’Kane CY, Roady TJ, Moran A, Scarry A, Carroll M, Volinsky L, Perez G, Patel P, Gabriel S, Lennon NJ, Madoff LC, Brown C, Smole SC (2021). Performance and implementation evaluation of the Abbott BinaxNOW rapid antigen test in a high-throughput drive-through community testing site in Massachusetts. J. Clin. Microbiol..

[16] Pilarowski G, Lebel P, Sunshine S, Liu J, Crawford E, Marquez C, Rubio L, Chamie G, Martinez J, Peng J, Black D, Wu W, Pak J, Laurie MT, Jones D, Miller S, Jacobo J, Rojas S, Rojas S, Nakamura R, Tulier-Laiwa V, Petersen M, Havlir DV, Consortium C, DeRisi J (2021). Performance characteristics of a rapid SARS-CoV-2 antigen detection assay at a public plaza testing site in San Francisco. J Infect Dis..

[17] Pollock, N. R., Tran, K., Jacobs, J. R., Cranston, A. E., Smith, S., O’Kane, C. Y., Roady, T. J., Moran, M., Scarry, A., Carroll, M., Volinsky, L., Perez, G., Patel, P., Gabriel, S., Lennon, N. J., Madoff, L. C., Brown, C. & Smole, S. C. Performance and operational evaluation of the Access Bio CareStart rapid antigen test in a high-throughput drive-through community testing site in Massachusetts. *MedRxiv*. (2021). Pre-print: 10.1101/2021.03.07.21253101v1.full-text. Accessed 5 April 2021. 10.1093/ofid/ofab243 PMC8244626 34250188

[18] Smith RL, Gibson LL, Martinez PP, Ke R, Mirza A, Conte M, Gallagher N, Conte A, Wang L, Fredrickson R, Edmonson DC, Baughman ME, Chiu KK, Choi H, Jensen TW, Scardina KR, Bradley S, Gloss SL, Reinhart C, Yedetore J, Owens AN, Broach J, Barton B, Lazar P, Henness D, Young T, Dunnett A, Robinson ML, Mostafa HH, Pekosz A, Manabe YC, Heetderks WJ, McManus DD, Brooke CB (2021). Longitudinal assessment of diagnostic test performance over the course of acute SARS-CoV-2 infection. medRxiv.

[19] Kilic A, Hiestand B, Palavecino E (2021). Evaluation of performance of the BD Veritor SARS-CoV-2 chromatographic immunoassay test in COVID-19 symptomatic patients. J. Clin. Microbiol..

[20] Okoye NC, Barker AP, Curtis K, Orlandi RR, Snavely EA, Wright C, Hanson KE, Pearson LN (2021). Performance characteristics of BinaxNOW COVID-19 antigen card for screening asymptomatic individuals in a university setting. J. Clin. Microbiol..

[21] FDA. Coronavirus (COVID-19) Update: FDA Continues to Advance Over-the Counter and Other Screening Test Development. U S Food and Drug Administration. (2021). Press Release, Published online: March 31, 2021.

[22] Healthcare T. Stop the Spread testing sites (2022). https://www.transformativehccom/stop-the-spread/. Accessed 27 Sept 2022.

[23] Epidemiologists CCoSaT. Update to the standardized surveillance case definition and national notification for 2019 novel coronavirus disease (COVID-19). Infectious Disease Committee. (2020). Accessed online: https://cdn.ymaws.com/www.cste.org/resource/resmgr/ps/positionstatement2020/Interim-20-ID-02_COVID-19.pdf

[24] Clinical Research Sequencing Platform (CRSP) LatBIoMaH. CRSP SARS-CoV-2 Real-time Reverse Transcriptase (RT)-PCR Diagnostic Assay. https://www.fdagov/media/139858/download

[25] FDA. Emergency Use Authorization (EUA) Summary: CRSP SARS-CoV-2 Real-Time Reverse Transcriptase (RT)-PCR Diagnostic Assay. (2022). https://www.fda.gov/media/146499/download. Accessed 28 Sept 2022

[26] Diagnostics A. CareStart COVID-19 Antigen: Rapid Diagnostic Test for the Detection of SARS-CoV-2 Antigen (2020). Accessed online: https://www.accessbiodiagnosticsnet/wp-content/uploads/2021/02/EUA-accessbio-CareStart-Antigen-ifupdf. Instructions for Use Under Emergency Use Authorization.

[27] Jaafar, R., Aherfi, S., Wurtz, N., Grimaldier, C., Hoang, T. V., Colson, P., Raoult, D. & La Scola, B. Correlation Between 3790 Quantitative Polymerase Chain Reaction–Positives Samples and Positive Cell Cultures, Including 1941 Severe Acute Respiratory Syndrome Coronavirus 2 Isolates. Clinical Infectious Diseases. (2020). Correspondence. 10.1093/cid/ciaa1491 PMC7543373 32986798

[28] Hernaez R, Thrift AP (2017). High negative predictive value, low prevalence, and spectrum effect: Caution in the interpretation. Clin. Gastroenterol. Hepatol..

[29] Syal K (2021). Guidelines on newly identified limitations of diagnostic tools for COVID-19 and consequences. J. Med. Virol..

[30] Dryden-Peterson S, Velasquez GE, Stopka TJ, Davey S, Lockman S, Ojikutu BO (2021). Disparities in SARS-CoV-2 testing in Massachusetts during the COVID-19 pandemic. JAMA Netw. Open.

[31] Yanes-Lane M, Winters N, Fregonese F, Bastos M, Perlman-Arrow S, Campbell JR, Menzies D (2020). Proportion of asymptomatic infection among COVID-19 positive persons and their transmission potential: A systematic review and meta-analysis. PLoS ONE.

[32] Perez-Garcia F, Romanyk J, Gomez-Herruz P, Arroyo T, Perez-Tanoira R, Linares M, Perez Ranz I, Labrador Ballestero A, Moya Gutierrez H, Ruiz-Alvarez MJ, Cuadros-Gonzalez J (2021). Diagnostic performance of CerTest and Panbio antigen rapid diagnostic tests to diagnose SARS-CoV-2 infection. J. Clin. Virol..

[33] Bullard J, Dust K, Funk D, Strong JE, Alexander D, Garnett L, Boodman C, Bello A, Hedley A, Schiffman Z, Doan K, Bastien N, Li Y, Van Caeseele PG, Poliquin G (2020). Predicting infectious SARS-CoV-2 from diagnostic samples. Clin. Infect. Dis..

[34] Rhee C, Kanjilal S, Baker M, Klompas M (2021). Duration of severe acute respiratory syndrome Coronavirus 2 (SARS-CoV-2) infectivity: When is it safe to discontinue isolation?. Clin. Infect. Dis..

[35] Levine-Tiefenbrun M, Yelin I, Katz R, Herzel E, Golan Z, Schreiber L, Wolf T, Nadler V, Ben-Tov A, Kuint J, Gazit S, Patalon T, Chodick G, Kishony R (2021). Initial report of decreased SARS-CoV-2 viral load after inoculation with the BNT162b2 vaccine. Nat. Med..

[36] Mina MJ, Peto TE, Garcia-Finana M, Semple MG, Buchan IE (2021). Clarifying the evidence on SARS-CoV-2 antigen rapid tests in public health responses to COVID-19. Lancet.

[37] Smith RL, Gibson LL, Martinez PP, Ke R, Mirza A, Conte M, Gallagher N, Conte A, Wang L, Fredrickson R, Edmonson DC, Baughman ME, Chiu KK, Choi H, Jensen TW, Scardina KR, Bradley S, Gloss SL, Reinhart C, Yedetore J, Owens AN, Broach J, Barton B, Lazar P, Henness D, Young T, Dunnett A, Robinson ML, Mostafa HH, Pekosz A, Manabe YC, Heetderks WJ, McManus DD, Brooke CB (2021). Longitudinal assessment of diagnostic test performance over the course of acute SARS-CoV-2 infection. J. Infect. Dis..

[38] Zhou Y, O’Leary TJ (2021). Relative sensitivity of anterior nares and nasopharyngeal swabs for initial detection of SARS-CoV-2 in ambulatory patients: Rapid review and meta-analysis. PLoS ONE.

[39] Lee RA, Herigon JC, Benedetti A, Pollock NR, Denkinger CM (2021). Performance of saliva, oropharyngeal swabs, and nasal swabs for SARS-CoV-2 molecular detection: A systematic review and meta-analysis. J. Clin. Microbiol..

[40] Soni A, Herbert C, Lin H, Pretz C, Stamegna P, Orwig T, Wright C, Tarrant S, Behar S, Suvarna T, Schrader S, Harman E, Nowak C, Kheterpal V, Rao LV, Cashman L, Orvek E, Ayturk D, Lazar P, Wang Z, Barton B, Achenbach CJ, Murphy RL, Robinson M, Manabe Y, Wang B, Pandey S, Colubri A, Oa Connor L, Lemon SC, Fahey N, Luzuriaga KL, Hafer N, Heetderks W, Broach J, McManus DD (2022). Performance of screening for SARS-CoV-2 using rapid antigen tests to detect incidence of symptomatic and asymptomatic SARS-CoV-2 infection: Findings from the Test Us at Home prospective cohort study. medRxiv.

[41] Trombetta BA, Kandigian SE, Kitchen RR, Grauwet K, Webb PK, Miller GA, Jennings CG, Jain S, Miller S, Kuo Y, Sweeney T, Gilboa T, Norman M, Simmons DP, Ramirez CE, Bedard M, Fink C, Ko J, De Leon Peralta EJ, Watts G, Gomez-Rivas E, Davis V, Barilla RM, Wang J, Cunin P, Bates S, Morrison-Smith C, Nicholson B, Wong E, El-Mufti L, Kann M, Bolling A, Fortin B, Ventresca H, Zhou W, Pardo S, Kwock M, Hazra A, Cheng L, Ahmad QR, Toombs JA, Larson R, Pleskow H, Luo NM, Samaha C, Pandya UM, De Silva P, Zhou S, Ganhadeiro Z, Yohannes S, Gay R, Slavik J, Mukerji SS, Jarolim P, Walt DR, Carlyle BC, Ritterhouse LL, Suliman S (2021). Evaluation of serological lateral flow assays for severe acute respiratory syndrome coronavirus-2. BMC Infect. Dis..

[42] Earnest R, Uddin R, Matluk N, Renzette N, Turbett SE, Siddle KJ, Loreth C, Adams G, Tomkins-Tinch CH, Petrone ME, Rothman JE, Breban MI, Koch RT, Billig K, Fauver JR, Vogels CBF, Bilguvar K, De Kumar B, Landry ML, Peaper DR, Kelly K, Omerza G, Grieser H, Meak S, Martha J, Dewey HB, Kales S, Berenzy D, Carpenter-Azevedo K, King E, Huard RC, Novitsky V, Howison M, Darpolor J, Manne A, Kantor R, Smole SC, Brown CM, Fink T, Lang AS, Gallagher GR, Pitzer VE, Sabeti PC, Gabriel S, MacInnis BL, Tewhey R, Adams MD, Park DJ, Lemieux JE, Grubaugh ND, New England Variant Investigation T (2022). Comparative transmissibility of SARS-CoV-2 variants Delta and Alpha in New England, USA. Cell Rep. Med..

